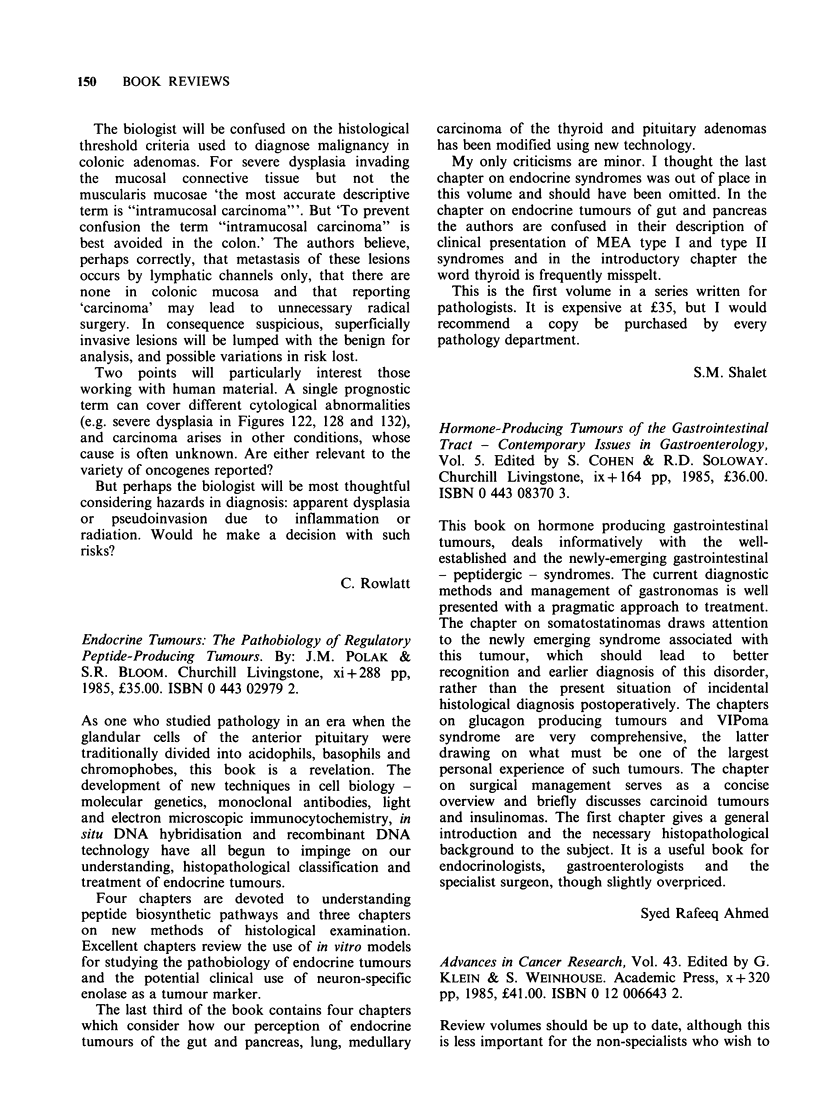# Hormone-Producing Tumours of the Gastrointestinal Tract - Contemporary Issues in Gastroenterolgy

**Published:** 1986-01

**Authors:** Syed Rafeeq Ahmed


*Hormone-Producing Tumours of the Gastrointestinal Tract – Contemporary Issues in Gastroenterology,* Vol. 5. Edited by S. COHEN & R.D. SOLOWAY. Churchill Livingstone, ix+164 pp, 1985, £36.00. ISBN 0 443 08370 3.

This book on hormone producing gastrointestinal tumours, deals informatively with the well-established and the newly-emerging gastrointestinal – peptidergic – syndromes. The current diagnostic methods and management of gastronomas is well presented with a pragmatic approach to treatment. The chapter on somatostatinomas draws attention to the newly emerging syndrome associated with this tumour, which should lead to better recognition and earlier diagnosis of this disorder, rather than the present situation of incidental histological diagnosis postoperatively. The chapters on glucagon producing tumours and VIPoma syndrome are very comprehensive, the latter drawing on what must be one of the largest personal experience of such tumours. The chapter on surgical management serves as a concise overview and briefly discusses carcinoid tumours and insulinomas. The first chapter gives a general introduction and the necessary histopathological background to the subject. It is a useful book for endocrinologists, gastroenterologists and the specialist surgeon, though slightly overpriced.

Syed Rafeeq Ahmed